# Psychological Symptoms During the Two Stages of Lockdown in Response to the COVID-19 Outbreak: An Investigation in a Sample of Citizens in Northern Spain

**DOI:** 10.3389/fpsyg.2020.01491

**Published:** 2020-06-18

**Authors:** Naiara Ozamiz-Etxebarria, Nahia Idoiaga Mondragon, María Dosil Santamaría, Maitane Picaza Gorrotxategi

**Affiliations:** ^1^Department of Developmental and Educational Psychology, University of the Basque Country UPV/EHU, Leioa, Spain; ^2^Department of Research and Diagnostic Methods in Education, University of the Basque Country UPV/EHU, Leioa, Spain; ^3^Department of Didactics and School Organization, University of the Basque Country UPV/EHU, Leioa, Spain

**Keywords:** stress, anxiety, depression, lockdown, COVID-19

## Abstract

Spain has been in a state of emergency since 14th March due to the COVID-19 crisis. This state of emergency means that the population must comply with strict rules such as lockdown (confinement to their homes except for essential trips) and social distancing. The aim of this study was to examine the psychological state of the general population in a sample recruited in Northern Spain. Sociodemographic and psychological data were gathered, assessing variables such as stress, anxiety, and depression. A questionnaire was administered at the beginning of the lockdown and three weeks later. The sample was recruited using an online questionnaire by means of a non-probabilistic snowball sampling methodology. A total of 1,933 people participated in this study. The results reveal that more than a quarter of the participants have reported symptoms of depression (27.5%), anxiety (26.9%) and stress (26.5%) and as the time spent in lockdown has progressed, psychological symptoms have risen. In relation to gender, data indicate that men have higher levels of depression than women, and similar levels of anxiety and stress. Greater symptomatology has also been found among the younger population and in people with chronic diseases. We discuss the need to continue carrying out these types of studies to prevent and treat psychological problems that could emerge amidst this pandemic.

## Introduction

In December 2019, an outbreak of new coronavirus pneumonia emerged in Wuhan (Hubei, China) (Chen et al., 2020). In early 2020, coronavirus disease (COVID-19) began to spread, firstly throughout China, and then rapidly throughout the world, with Europe in general and some countries in particular like Spain becoming strongly affected by contagion and deaths caused by the pandemic (De Giorgio, 2020). This rapid and unprecedented pandemic has created significant mental health problems (Torales et al., 2020) such as stress, anxiety and, depression for both medical professionals and the general population alike (Liu S. et al., 2020).

In the Basque Autonomous Community, a region located in Northern Spain, the coronavirus alarm was triggered in March 2020. In this region of 2,167,707 inhabitants, the first case was detected on February 28, after which there has been a rapid rise in cases. On 12th March the Basque Government temporarily suspended classes in all educational centers from nurseries to the University. On March 13th the Council of the Basque Government declared a health emergency and on March 14th the Spanish Government declared the state of emergency and ordered a lockdown in which all citizens were confined to their homes, creating an unprecedented situation (Department of Health of the Basque Government, 2020).

When this research began on 11th March 2020, 225 cases and 11 deaths had been confirmed in the Basque Autonomous Community. On March 18, 1,190 cases, 50 deaths and 18 recovered. On April 2, 7,827 cases, 444 deaths and 367 recovered. And finally on April 12th, at the end of this study, 11,018 cases, 831 deaths and 1,209 recovered had been confirmed on that territory (Basque Governement, 2020).

Beyond the medical risks, the psychological and social impact of this pandemic is indisputable. A number of previous research studies have focused on understanding how society defines the origin and impact of emerging infectious diseases, underlining the importance of being able to cope with such crises on an emotional level (Idoiaga et al., 2017a).

Although COVID-19 has emerged very recently, due to the unprecedented nature of this pandemic several studies have already been carried out to examine its consequences, primarily in China but also in Europe (Fagiolini et al., 2020; Porcheddu et al., 2020; Qiu et al., 2020). Research from China, the first affected country, suggests that the fear of this pandemic can lead to mental illness such as stress disorders, anxiety, depression, somatization and behaviors such as increased alcohol and tobacco consumption (Shigemura et al., 2020). Moreover, the application of strict lockdown measures in that country is affecting many aspects of people’s lives, triggering a wide variety of psychological problems, such as panic disorder, anxiety, and depression (Qiu et al., 2020).

A study carried out between 31st January and 2nd February 2020 with 1,210 people in 194 cities of China, administered the Depression, Anxiety and Stress Scale (DASS-21). The aim of this study was to conduct an online survey using snowball sampling techniques to better understand (among other variables) the levels of psychological impact, anxiety, depression and stress in the early stages of the COVID-19 outbreak. The results revealed that 16.5% of participants showed moderate to severe depressive symptoms; 28.8% showed moderate to severe anxiety symptoms; and 8.1% reported moderate to severe stress levels (Moghanibashi, 2020; Wang et al., 2020a). Moreover, poor health was significantly associated with a greater psychological impact and higher levels of stress, anxiety, and depression (Wang et al., 2020a).

It is therefore clear that the COVID-19 pandemic represents a source of stress due to uncertainty and lack of knowledge (Craske and Stein, 2017; Yenan et al., 2020) and financial difficulties (Tran et al., 2020b). However, we should not assume that this pandemic affects the entire population equally at either a medical level (CDC, 2020; Garg, 2020) or a psychological level (Liu S. et al., 2020; Yeen and Zhao, 2020). From a medical perspective, COVID-19 is particularly severe for the elderly and for people with chronic diseases (Wenjun et al., 2020), although serious adverse effects have also been found in children (Licciardi et al., 2020).

Nonetheless, from a psychological perspective, young adults have shown high levels of psychological symptoms in response to the COVID-19 outbreak (Lai et al., 2020). In fact, in a nationwide survey of psychological distress in China with more than 52,000 participants, people between 18 and 30 years and those above 60 presented the highest posttraumatic distress index scores (Qiu et al., 2020). These high levels of distress among young people could be due to the fact that they tend to gather a large amount of information from social media, which could easily trigger stress (Bao et al., 2020). In contrast, distress in older people could be explained by the fact that this population is suffering from the highest mortality rates as a result of COVID-19. During the COVID-19 pandemic, psychiatric patients were prone to develop anxiety and depression due to lack of access to psychiatric care (Hao et al., 2020). Workers were prone to adverse mental health due to perceived risk of contracting COVID-19 at the workplace (Tan et al., 2020). Healthcare workers experienced high levels of anxiety due to concerns about spreading COVID-19 to their family members (Chew et al., 2020).

Further, lockdown is also a very important factor in psychological well-being, since previous studies of isolation similar to that being experienced in the current health crisis found that younger age and gender predicted a negative psychological impact of the lockdown (Taylor et al., 2008; Altena et al., 2020). In addition, a recent study has demonstrated that post-traumatic stress symptoms in Wuhan residents following the outbreak of COVID-19 were particularly high among women under 35 years of age and in those people who had reported watching the news three times a day (Gao W. et al., 2020; Huang and Zhao, 2020).

Therefore, and as previously noted, gender could be another variable to be considered in the psychological response to the pandemic. In fact, much of the research has shown that women appear to present more severe symptoms of depression, anxiety and distress in comparison with men (Lai et al., 2020; Liu N. et al., 2020; Qiu et al., 2020). However, another recent research study in China that analyzed anxiety disorder, depressive symptoms, and sleep quality found that one in three participants showed anxiety disorders, but mood states did not differ between males and females during the COVID-19 epidemic, which contrasts with the findings of previous research showing that women are more likely to suffer from anxiety when compared with men (Huang and Zhao, 2020).

Finally, recent studies have also shown that this increased anxiety resulting from COVID-19 could be particularly prevalent among people with a history of psychiatric problems (Hao et al., 2020). Moreover, some researchers have also pointed out that people with chronic diseases are expected to have higher levels of psychological symptoms (Applegate and Ouslander, 2020), since COVID-19 tends to be more severely manifest in those people with multiple underlying diseases (Dong et al., 2020).

In any health crisis, fear, uncertainty, and stigmatization are common and it is therefore important to apply appropriate medical and mental health interventions (Xiang et al., 2020). Thus, in an international public health emergency of the sort that we are currently experiencing, it is important to investigate the psychological impact of the pandemic among specific populations in order to develop tailored strategies aimed at reducing the symptoms that could occur during the crisis (Wang et al., 2020a).

Therefore, the present study aims to measure the levels of stress, anxiety, and depression in a sample of people from a region highly affected by COVID-19—the Basque Autonomous Community in Northern Spain. The general population is expected to have symptoms of stress, anxiety, and depression from the situation generated by the COVID-19 crisis. What is more, those stress, anxiety and depression levels are expected to increase as the period of lockdown progresses (Brooks et al., 2020; Sha et al., 2020) since confinement leads to these types of psychological problems (Cava et al., 2005). In order to analyze this possible progression, stress, anxiety and depression were measured in two stages: (1) the time at which the government declared the state of emergency, and (2) at 2–3 weeks after lockdown.

It was also anticipated that the levels of anxiety, depression and stress will not be homogeneous across society, and that there will therefore be specific and significant differences between groups. In particular, potential differences will be analyzed according to gender, age and previous chronic diseases with women expected to present higher levels of psychological symptoms than men, as indicated in several studies conducted in China. Additionally, younger people and those with a prior history of chronic diseases are expected to show higher levels of stress, anxiety, and depression than the general population.

## Materials and Methods

### Participants

A total of 1,993 people from the Autonomous Community of the Basque Country aged between 18 and 82 years participated in the study (*M* = 33.80, *SD* = 16.65), 55.5% (*n* = 1,106) were aged between 18 and 30 years, 31.9% (*n* = 636) between 31 and 59 years, and 12.6% (*n* = 251) were over 60 years old. Of the sample 79.5% (*n* = 1,584) were female, 20.1% (*n* = 401) were male and 0.4% (*n* = 8) were other. In addition, 17.2% (*n* = 343) of the sample reported having a chronic disease and 82.8% (*n* = 1,650) reported having no disease. Finally, the questionaires were completed in two periods of the health crisis, 1,112 (55.8%) of the participants completed the questionnaire between the 11th and 18th of March and 881 (44.2%) between the 2nd and 12th of April.

### Measures and Instruments

In the *ad hoc* survey carried out to gather sociodemographic data of the participants, which adopted a closed answer format, the participants were asked about sex, age, province, date of completion of the questionnaire and whether or not they had a chronic illness. Subsequently, the participants were categorized into three age groups (18–35, 36–59 and over 60 years).

The Depression Anxiety and Stress Scale—21 (*DASS-21*, Ruiz et al., 2017) was administered. The DASS-21 scale is composed of 21 Likert-type items that represent 3 factors: Depression (Items: 3, 5, 10, 13, 16, 17, and 21), Anxiety (Items: 2, 4, 7, 9, 15, 19, and 20) and Stress (Items: 1, 6, 8, 11, 12, 14, and 18). The response options for this scale were: 0: It did not happen to me; 1: It happened to me a little, or for some of the time; 2: It happened to me a lot, or for a good part of the time; and 3: It happened to me a lot, or most of the time, using questions such as: “I overreacted in certain situations,” “I have felt uneasy.” As each subscale of the *DASS-21* consists of 7 items and the total values of anxiety, depression and stress are calculated by the sum of the values of each of the items. Therefore, the total value achievable on each subscale is within the range of scores 0–21. In relation to the reliability of the scale the Cronbach’s alpha coefficient for this study varied depending on the factor: for depression, α = 0.88, for anxiety, α = 0.81 and for stress, α = 0.85. DASS-21 was used to measure mental health of the general population (Wang et al., 2020b) and healthcare workers (Tan et al., 2020) during the COVID-19 pandemic.

### Procedure

The first step was to secure permission from the university ethics committee to carry out this study. The approval of the Ethics Committee of the UPV/EHU was obtained [M10/2020/055]. All the people participated on a voluntary basis, received information about the procedure of the investigation and gave their consent before participating in the study. Therefore, the Ethics Committee, in compliance with the Helsinki Declaration of the World Medical Association, gave their approval for the procedure followed here. The sample was recruited by non-probabilistic snowball sampling. Once the Google Forms questionnaire had been created, it was disseminated through virtual platforms, social networks, and through corporate emails sent out by the researchers. The first stage of the study was defined as the week in which the state of emergency was declared in Spain (from 3 days before to 4 days after), that is, from the 11th to the 18th of March. The second stage of the study took place 2–3 weeks later, when people had been in lockdown for 20 days, that is, from the 2nd to the 12th of April. A total of 2,200 people responded. Once the database had been analyzed using the Excel program, an analysis of the response items was carried out and a pattern of non-response of more than 50% was observed in some subjects, the rest of the participants answered all the questions. Therefore, we decided to remove these subjects from the sample, leaving a total of 1,993 participants in the work matrix. Of this final 1,993, data from 8 respondents identifying like others in their gender were not used to show gender differences, as they were not a sufficient population to conduct the relevant analyses below.

### Data Analysis

The data were imported from the Excel calculation matrix into the statistical program SPSS v.25 to perform the appropriate analyses. Before proceeding to explain the relevant analyses, the assumptions of normality and homocedasticity of variances were checked to decide on the use of parametric or non-parametric tests. Specifically, the Kolmogorov-Smirnov statistical test indicated that the data did not follow a normal distribution in all variables of the study. However, it should be noted that there is support in the scientific literature for the robustness of parametric tests even when there is a violation of the assumptions of normality and homocedasticity, taking into account the asymmetry and kurtosis of the data, which in most variables did not exceed 1. With regard to the analyses performed, it should be noted that descriptive analyses were carried out to study the frequencies of the dependent and independent variables in the sample. Subsequently, comparative analyses were carried out between the dependent and independent variables in two groups of the sample (use of total scores; 0–21), specifying the interval coefficients and the effect sizes of the family of standardized mean differences with Cohen’s (1988). Finally, an ANOVA was carried out in order to observe the differences of the dependent variables in the case of having an independent variable in three groups (age). For the difference between the groups, Bonferroni’s tests between groups were used.

## Results

### Descriptive Analysis of the Sample

Of the participants in this the study, 27.5% reported symptoms of depression, 26.9% anxiety and 26.5% stress. In relation to the symptoms studied, Table 1 displays the percentages of respondents who did and did not report suffering from any type of symptomatology.

**TABLE 1:** Frequencies and percentages of symptoms studied as a function of independent variables.

Depression	No	Yes	Anxiety	No	Yes	Stress	No	Yes
**Sex**								
Men	69.8%	30.2%	Men	72.8%	27.2%	Men	72.6%	27.4%
Women	73.3%	26.7%	Women	73.2%	26.8%	Women	74%	26%
**Age**								
18–30	68.4%	31.6%	18–30	69.7%	30.3%	18–30	69.9%	30.1%
31–59	76.7%	23.3%	31–59	74.1%	25.9%	31–59	74.3%	25.7%
<60	81.3%	18.7%	<60	86.9%	13.1%	<60	88.8%	11.2%
**Period of the health crisis**								
>18 March	78.2%	21.8%	>18 March	77%	23%	>18 March	78.1%	21.9%
<2 April	65.6%	34.4%	<2 April	68.6%	31.4%	<2 April	68.1%	31.9%
**Chronic illness**								
Yes	68.2%	31.8%	Yes	68.5%	31.5%	Yes	70.3%	29.7%
No	73.6%	26.4%	No	74.2%	25.8%	No	74.4%	25.6%

### Comparison of Means Between the Dependent and Independent Study Variables

Table 2 shows significant differences between men and women in relation to depressive symptomatology, *t* (571) = 2.17, *p* = 0.02, *d*_Cohen_ = 0.13, with a small effect size. Women show a lower mean (*M* = 3.26; *SD* = 3.84) than men (*M* = 3.77; *SD* = 4.30). Likewise, as expected, there are differences in the symptoms of depression, anxiety, and stress according to the time point at which the data were collected from the sample; depression, *t* (1991) = 7.32, *p* = 0.001, *d*_Cohen_ = 0.49, anxiety *t* (1991) = 3.95, *p* = 0.001, *d*_Cohen_ = 0.17 and stress *t* (1991) = 6.92, *p* = 0.001, *d*_Cohen_ = 0.31 Also, a moderate effect size on depression was found, followed by stress and with a small effect size anxiety.

**TABLE 2:** Types of symptoms according to gender.

		*n*	*M*	*SD*	*t*	*p*	95% CI	*d*_Cohen_
Depression	Women	1584	3.26	3.84	–2.31	0.021*	−0.94, −0.78	0.13
	Men	401	3.77	4.30				
Anxiety	Women	1584	2.51	3.11	–1.11	0.266	−0.58, 0.15	0.06
	Men	401	2.71	3.52				
Stress	Women	1584	5.23	4.15	–1.22	0.225	−0.77, 0.20	0.07
	Men	401	5.52	4.48				
Depression	11–18 March	1.112	2.80	3.57	–7.32	0.001***	−1.63, −0.94	0.49
	2–12 April	881	4.71	4.25				
Anxiety	11–18 March	1.112	2.30	2.93	–3.95	0.001***	−0.85, −0.29	0.17
	2–12 April	881	2.86	3.47				
Stress	11–18 March	1.112	4.71	3.95	–6.92	0.001***	−1.67, −0.93	0.31
	2–12 April	881	6.01	4.44				
Depression	C.D Yes	343	3.81	4.38	2.36	0.018*	0.09, 1.00	–0.13
	C.D No	1.650	3.26	3.83				
Anxiety	C.D Yes	343	3.01	3.77	2.97	0.003**	0.19, 0.93	–0.16
	C.D No	1.650	2.46	3.05				
Stress	C.D Yes	343	5.58	4.47	1.40	0.162	−0.14, 0.84	–0.07
	C.D No	1.650	5.29	4.17				

* *p* < 0.05; ** *p* < 0.01; *** *p* < 0.001. C.D = Chronic disease.

Finally, we analyzed differences between symptoms based on whether participants reported suffering from a chronic disease. The results of these analyses are shown in Table 2. In relation to depression, patients with chronic disease show significant differences, *t* (1991) = 2.36, *p* = 0.018, *d*_Cohen_ = 0.13. Those who showed higher mean scores (*M* = 3.81; *SD* = 4.38) were those who were not chronically ill (*M* = 3.26; *SD* = 3.83). A similar pattern of results was observed for anxiety, *t* (1991) = 2.97, *p* = 0.003, *d*_Cohen_ = 0.16, chronic patients showing higher mean scores (*M* = 3.01, *SD* = 3.77) than non-chronic patients (*M* = 2.46, *SD* = 3.05).

An unifactorial ANOVA was conducted to analyze the variability of the studied symptoms according to age. This analysis revealed significant differences between the age groups for depression, anxiety and stress. The largest effect size among the three age categories was found when measuring stress, *F*(2, 1990) = 30.01, *p* = 0.001, *η*^2^ = 0.29. However, in order to confirm which of the comparisons between the age groups yielded a significant difference in terms of symptomatology, *post hoc* analysis was conducted using a Bonferroni test (given the assumption of equal variances). The results indicate that when comparing the 18–30 and 31–59 age categories, significant differences only emerged for the depression scores. Further, significant differences were found between the 18–30 and over 60 years age groups for the three symptoms. Similar differences also emerged between the 31–59 and over 60 years age groups (see Table 3).

**TABLE 3:** Types of symptomatology according to age and *post hoc* comparisons.

DV	Age (years)	*n*	M	SD	*F*	*p*	*η*^2^	*Post-hoc*
Depression	18–30	1106	3.84	4.15	21.61	0.001***	0.21	1–2
	31–59	636	2.95	3.73				1–3
	60–82	251	2.30	3.97				2–3
Anxiety	18–30	1106	2.84	3.35	21.05	0.001***	0.21	1–2
	31–59	636	2.49	3.21				1–3
	60–82	251	1.41	1.89				2–3
Stress	18–30	1106	5.66	4.34	30.01	0.001***	0.29	1–3
	31–59	636	5.36	4.16				2–3
	60–82	251	3.41	3.26				

* *p* < 0.05; ** *p* < 0.01; *** *p* < 0.001.

## Discussion

A number of the participants in this study appear to have shown levels of stress, anxiety and depression since the outbreak of COVID-19 in Northern Spain, as also found in several studies in China and Europe (Altena et al., 2020; Asmundson and Taylor, 2020; Gao J. et al., 2020; Sani et al., 2020; Wang et al., 2020a). Among the participants of this study, more than a quarter have reported symptoms of depression (27.5%), anxiety (26.9%) and stress (26.5%). Although there is a significant proportion of people with psychological symptoms, it should be stressed that these data provide more grounds for optimism than those found in other studies. For example, in an analysis of the psychological burden caused by SARS (Su et al., 2007) and even the COVID-19 in China (Huang and Zhao, 2020) it was found that one in three participants had anxiety disorders. The reasons for this higher symptomatology could lie in the fact that, in addition to the concerns about being infected, these previous studies were conducted in situations of prolonged and stringent lockdown measures.

Our findings have also shown that stress, anxiety and depression levels are higher when measured two-three weeks after starting the lockdown, since the participants who responded during the second phase appear to suffer more from these symptoms. This increase in symptomatology is of concern since it is not yet known how much longer the population will be in lockdown and it has been shown that confinement has a psychological impact on individuals (Brooks et al., 2020). For example, lockdown could lead to a lack of sufficient sunlight, which causes a fall in serotonin levels that is associated with emotional disorders such as anxiety and depression (Lambert et al., 2002). It is important to keep these data in mind because if the lockdown measures are kept in place over a long period of time, people may become psychologically disturbed, and could even suffer from problems such as post-traumatic stress disorder (Bao et al., 2020; Petzold et al., 2020).

In addition, the results show, as expected, that people with chronic diseases were more likely to suffer from symptoms of anxiety and depression. These results are consistent with research showing that people with severe illness or multiple illnesses suffer from higher levels of psychological symptoms amid this health crisis (Dong et al., 2020). Therefore, any psychological containment plan should consider these individuals and provide them with specifically adapted tools and strategies to cope—both physically and psychologically—with COVID-19.

In relation to gender, our findings are particularly striking, since unlike the results found in other research studies, our data indicate that men have higher levels of depression than women, and similar levels of anxiety and stress (Lai et al., 2020; Liu N. et al., 2020). These results also run counter to other published works suggesting that women are being hit harder and will suffer more from the consequences of this COVID-19 crisis (Guo et al., 2016; Gao W. et al., 2020). The reason for these discrepant results could be that, as is usually the case in psychosocial studies in Spain (INE, 2016), more women participated in this study, and the men who did agree to take part may have done so due to their feelings of apprehension regarding the crisis.

In terms of age, young adults (18–30) and adults aged between 31 and 59 years have higher levels of stress, anxiety and depression in comparison with the elderly (60–82 years). In fact, people with the highest levels of anxiety and depression are the young adults in the 18–30-year age range. These findings are, in part, consistent with those of studies conducted in China where young adults reported a higher prevalence of depressive and anxiety symptoms (Yeen and Zhao, 2020). This apparently higher symptomatology among young people could be caused by the large amount of information that they receive from social media, including fake news, which could easily trigger stress (Bao et al., 2020; Huang and Zhao, 2020; Kumar and Somani, 2020). Moreover, given that the younger participants of our sample were mostly students, this stress could also be associated with the added burden experienced by young students, given their need to adapt to the new educational context without face-to-face classes (Aracena et al., 1992; Martín, 2007; Vélez et al., 2010; Antúnez and Vinet, 2012). In this regard, whilst the educational institutions implemented online educational strategies from the beginning of this health crisis, it appears that these did not serve to reassure the youth in these moments of uncertainty. Therefore, if this young population is also considered to be vulnerable to emotional disorders, it will be vitally important that educational institutions put into place prevention and intervention programs aimed at reducing these levels of depression, anxiety and stress (Aracena et al., 1992; Cova et al., 2007).

The findings obtained in our sample also shows that the level of stress is equally high for young adults and adults. To be able to limit the risk of this symptomatology we should take into account that in previous pandemics it was found that the most frequent stressors in adults were the duration of lockdown measures, fear of being infected, frustration, boredom and inadequate information (Brooks et al., 2020). Other researchers have also found that lockdown could create post-traumatic stress in adults, particularly in relation to financial losses (Mihashi et al., 2009) and stigma (Wester and Giesecke, 2019).

Whilst the Spanish Government has implemented very stringent containment measures to prevent the further spread of the COVID-19 outbreak, our study highlights the importance of conducting research to investigate the way in which these measures could have a psychological impact on the population. Appropriate social intervention to promote psychological well-being should also be implemented, as pointed out by some studies in China (Huang and Zhao, 2020). To begin with, it would be advisable for the media to disseminate only accurate and reliably sourced information. It is important to manage the vast body of unfiltered information transmitted by the media and social networks (Bao et al., 2020). In fact, in Spain, alarming videos on COVID-2019 are circulating freely and are accessible to almost all individuals, particularly young people, which could also be a factor in their apparent psychological vulnerability. For all of these reasons, it is critical to ensure effective communication in order to avoid public health risks. Thus, in emergency situations such as the one we are currently experiencing, it is more important than ever for experts such as medical professionals and governing bodies to be prepared to transmit information to the public in an effective and direct manner (Sandman, 2003; Idoiaga et al., 2017b; Ruiz de Azúa et al., 2020; Tran et al., 2020a, b).

Moreover, psychological counseling should be made available an official public platform adapted to different target groups (Liu S. et al., 2020). Cognitive behavior therapy and mindfulness therapy are particularly useful to improve mental health during COVID-19 pandemic (Ho et al., 2020). In different countries, numerous psychiatric hospitals, psychological counseling centers and university psychology departments have set up specialized telephone lines to provide psychological counseling services (Bao et al., 2020; Fagiolini et al., 2020). Furthermore, it would also be important to provide specific aid to each target group, with particular emphasis not only on people with chronic illnesses but also young people.

In the Basque Country, psychological services were launched in the Basque Health Service (Osakidetza) from the moment that the cases of COVID-19 began to increase. These services not only attend to patients and their families but also to all of the primary care professionals working in the hospitals (particularly emergency services, ICUs, respiratory and infectious services, and the Health and Emergency Council). The aim of this service is to detect, and if necessary, provide care and support for professionals in Osakidetza who have psychological/emotional disorders and who treat COVID-19 patients (Osakidetza, 2020). However, this aid service is only available for infected people and as this study has shown, lockdown itself can also cause serious deterioration in mental health, even in those people not infected.

As recommendations for the general public, we should, above all, highlight the importance of self-care and the need to balance free time with other activities, to monitor the amount of time spent watching the news or receiving information from the media, to maintain normal working hours, to rest as much as possible, to exercise regularly, to focus on the quality of sleep, and, particularly, to avoid paying too much attention to information about epidemics before going to sleep (Huang and Zhao, 2020).

In sum, this research contributes toward identifying the symptomatology shown by the population in Northern Spain at two different phases of the COVID-19 crisis. The increase in psychological symptoms forces reflection on the importance of taking preventive measures so that these symptoms do not worsen over time (Li et al., 2020). There are a number of factors that might play a role in the psychological state of the population. First, the time spent in lockdown is increasing, and this could lead to a worsening of the population’s mental health as there is great uncertainty about what may happen in the future. Second, the young adult population—although not a risk group for COVID-19 at a clinical level—is at risk from a psychological perspective since young people are suffering the most according to this study. Therefore, it is important to address their psychological needs and to provide them with specific attention, since in comparison with older people they are likely to have fewer tools to cope with this situation. The chronically ill population also requires attention, not only by addressing their physical health through social distancing, but also by addressing the psychological difficulties that they might be experiencing amid this pandemic. It is also important to mention that although this study was carried out in the north of Spain, the needs detected from the findings can be generalized to other populations since several studies have detected that the COVID-19 is generating psychological symptoms in the general population (Altena et al., 2020; Asmundson and Taylor, 2020; Gao J. et al., 2020; Sani et al., 2020; Wang et al., 2020a).

The study has some limitations that will have to be considered for future studies. One of the limitations of this study is that it is a non-probabilistic sample and a cross-sectional study. Besides, although information has been collected on chronic diseases, it has not been specified which chronic diseases and what level of severity the subjects have. This is an aspect that will have to be taken into account in future studies. Furthermore, other significant variables such as the level of education or income have not been collected. These variables would be very interesting to collect in a future study. In addition to these variables, it will be interesting to continue collecting data on stress, anxiety and depression in the population as new measures are being taken for people such as social distancing. Furthermore, the fear of new outbreaks may also be creating psychological symptoms in the population.

## Conclusion

In a sample recruited in Northern Spain, the present study explored the psychological status of people assessed at different stages of lockdown during the COVID-19 outbreak. Our findings reveal some of the variables that could contribute toward a worsening state of mental health in this new and unprecedented situation of tension and uncertainty. Therefore, it is important to monitor the state of mental health of the population in order to prevent and treat possible mental illnesses in the future.

## Data Availability Statement

The raw data supporting the conclusions of this article will be made available by the authors, without undue reservation.

## Ethics Statement

The studies involving human participants were reviewed and approved by the Ethics Committee for Research Related to Human Beings (CEISH) of the University of the Basque Country UPV/EHU [M10/2020/055]. The patients/participants provided their written informed consent to participate in this study.

## Author Contributions

NI, MP, and NO-E were involved in the conceptualization of the project and in the acquisition and analysis of the data. MD was involved in the interpretation of the data. All authors were involved in the drafting and revising of the work for intellectual content, provided approval for submission of the contents for publication, and agreed to be accountable for the accuracy and integrity of the project.

## Conflict of Interest

The authors declare that the research was conducted in the absence of any commercial or financial relationships that could be construed as a potential conflict of interest.
