# Corrigendum: Psychological Symptoms During the Two Stages of Lockdown in Response to the COVID-19 Outbreak: An Investigation in a Sample of Citizens in Northern Spain

**DOI:** 10.3389/fpsyg.2020.02116

**Published:** 2020-09-01

**Authors:** Naiara Ozamiz-Etxebarria, Nahia Idoiaga Mondragon, María Dosil Santamaría, Maitane Picaza Gorrotxategi

**Affiliations:** ^1^Department of Developmental and Educational Psychology, University of the Basque Country UPV/EHU, Leioa, Spain; ^2^Department of Research and Diagnostic Methods in Education, University of the Basque Country UPV/EHU, Leioa, Spain; ^3^Department of Didactics and School Organization, University of the Basque Country UPV/EHU, Leioa, Spain

**Keywords:** stress, anxiety, depression, lockdown, COVID-19

In the original article, we neglected to include the funder KideOn Research Group of the Basque Government, Ref.: IT1342-19 (A category).

The authors apologize for this error and state that this does not change the scientific conclusions of the article in any way. The original article has been updated.

